# Supplementary material for: Unveiling the detection dynamics of semiconductor nanowire photodetectors by terahertz near-field nanoscopy
Source: Light Sci Appl. 2020 Nov 19;9:189. doi: 10.1038/s41377-020-00425-1 (PMC7677312; doi:10.1038/s41377-020-00425-1)
Supplement: Supplementary file 1 — Supplemental material [file 41377_2020_425_MOESM1_ESM.pdf]

Supplementary Information

Unveiling the detection dynamics of semiconductor nanowire photodetectors by terahertz near-field nanoscopy

Eva A.A. Pogna,[1*] Mahdi Asgari, Valentina Zannier,[1] Lucia Sorba,[1] Leonardo Viti,[1] and Miriam S. Vitiello[1**]

[1] *NEST, CNR - Istituto Nanoscienze and Scuola Normale Superiore, Piazza San Silvestro 12, 56127, Pisa, Italy*

* eva.pogna@nano.cnr.it
** miriam.vitiello@sns.it

1. Electrical characterization

1.1 I-V curves

The Ohmic behavior of the electrical contacts, the efficacy of the gating and the lack of leakage through the dielectric has been verified by monitoring the source-drain current of the nanowires as a function of the source-drain bias for different values of the gate voltage V_G, as reported in Fig. S1 for NW2.

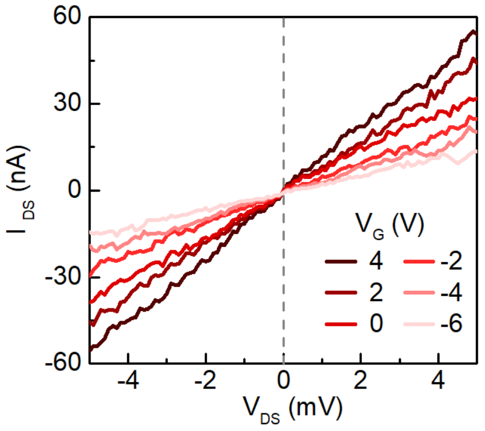

Figure S1: I-V curve of NW2 collected at different gate voltages V_G.

1.2 Determination of the Seebeck coefficient for NW1 and NW2 as function of gate bias

To extrapolate the Seebeck coefficient of the two samples we follow the same procedure described in Ref. 1. We use the Mott's formula:

$$S_e = -\frac{\pi^2 k_B^2}{3e} T \frac{1}{\sigma} \frac{d\sigma}{dE}\bigg|_{E=E_f} \qquad \text{(Eq. S1)}$$

where k_B is the Boltzmann constant, σ is the conductivity, T the temperature, e the electron charge and E_f the Fermi energy. We consider the conductivity in Fig. 1b (main text) in the linear transport regimes where we can assume constant mobility: μ^{NW1} = 740 cm^2 V^{-1} s^{-1} and μ^{NW2} = 505 cm^2 V^{-1} s^{-1}, see Figure S2a. Accordingly, in this regime, Eq. S1 can be simplified as follows:

$$S_e = -\frac{\pi^2 k^2}{3Cg} T \left(\frac{1}{\sigma} \frac{d\sigma}{dV_G} \right) \frac{dn_e}{dE_f} \qquad \text{(Eq. S2)}$$

where n_e is the carrier density and C_g is the capacitance between NW and gate normalized to the gated volume.

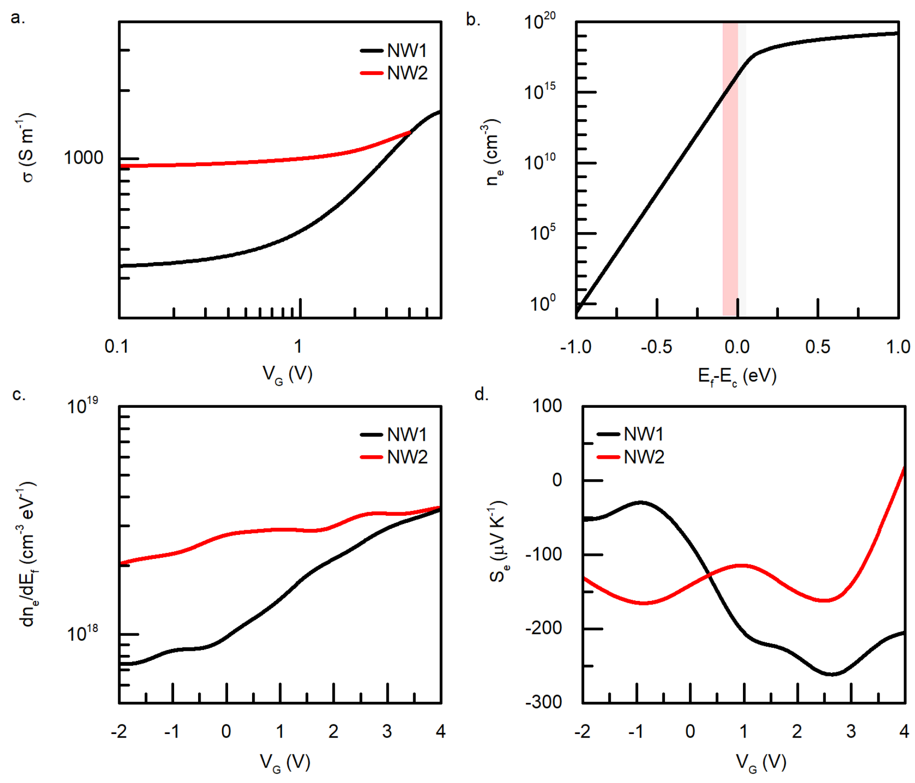

Figure S2: Seebeck coefficient estimation. (a) Conductivity as a function of gate voltage for NW1 and NW2 in log-log scale from Figure 1b; **(b)** Carrier density n_e of a 3D electron gas as a function of the Fermi level E_f compared to the energy of the conduction band minima E_c. Red and grey bands correspond to the carrier density ranges explored with V_G in the NW1 and NW2, respectively**. (c)** First-derivative of the function in (b) evaluated at the carrier densities corresponding to gate voltage V_G in NW1 (black line) and NW2 (red line). **(d)** Seebeck coefficient in NW1 (black line) and NW2 (red line) estimated assuming constant mobility.

The derivative $\frac{dn_e}{dE_f}$ is estimated considering the three-dimensional electron density of states $g(E)$ which is valid for NW radii larger than 20 nm and effective mass m*=0.023 m_e, where m_e is the electron mass in vacuum, see Fig. S2b. At every gate voltage V_G we evaluate n_e from the measured conductivity $\sigma = en_e\mu$, assuming a constant mobility. The Seebeck coefficient $S_e(n_e)$ in Fig. S2 is then evaluated from Eq. S2 considering $\frac{dn_e}{dE_f}$ at the carrier densities explored with the gate.

2. THz-nanoscopy

2.1 Spatial resolution and far-field background

In our nanoscopy experiments, the tip is operated in tapping mode with oscillation frequency Ω. The near-field signal depends non linearly on the tip-sample distance as it is shown in Figure S3. Increasing the demodulation order, the signal decreases and we observe the virtual tip sharpening effect[2].

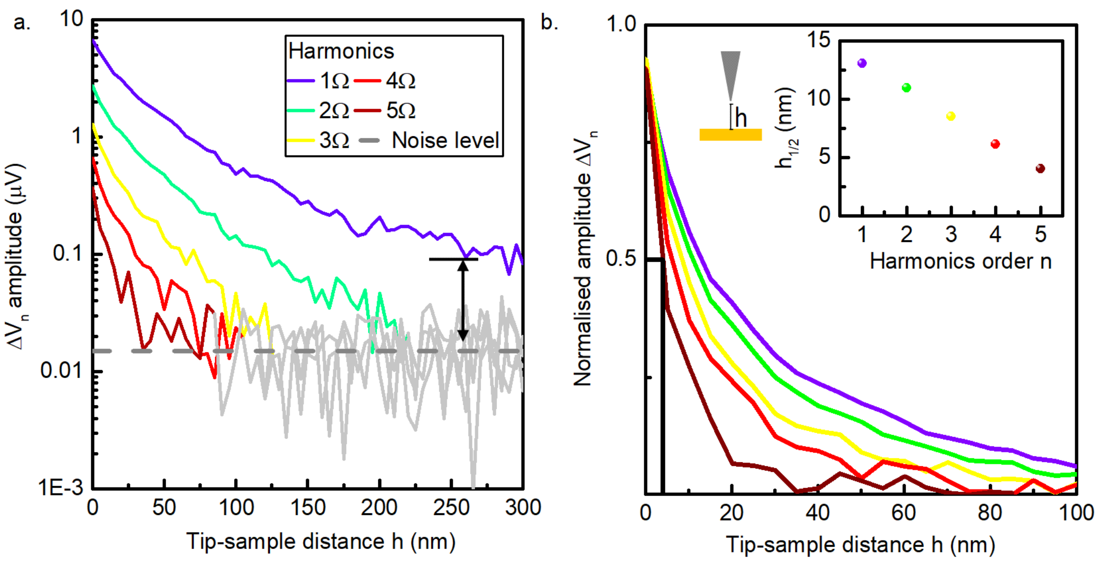

Figure S3: Approach curves at the different harmonics recorded on the InAs nanowire NW1 reported in semi-logarithm scale **(a)** and in linear scale **(b)** to calculate the distance $h_{1/2}$ (inset) at which the normalized signal drops to half of its maximum.

To find a good compromise between signal intensity and suppression of the background due to non-local scatterers, we chose the 2nd order to analyze the photocurrent maps, being *n*= 2

the lowest order Fourier component at which the signal reaches the noise level once approaching the tip from the sample. The decay distance $h_{1/2}$ at which the amplitude signal has decayed by a factor of 2, is also shown.

We characterize the signal amplitude s_n and lateral resolution Δx by analyzing the line profiles across the interface between the electrodes and the nanowire in the case of the photo-current signal and of the self-mixing signal induced by the scattered THz field.

We define the spatial resolution in analogy to other microscopy techniques as the full width at half maximum (FWHM) of the line spread function (LSF). The LSF can be calculated by taking the derivative of the line profile measured across an interface. The black dots in Figure S4 show the line profile across the source and nanowire interface, from which we extract the first derivative (in the inset), fit with Lorentzian functions with FWHM of 35 ± 2 nm, which

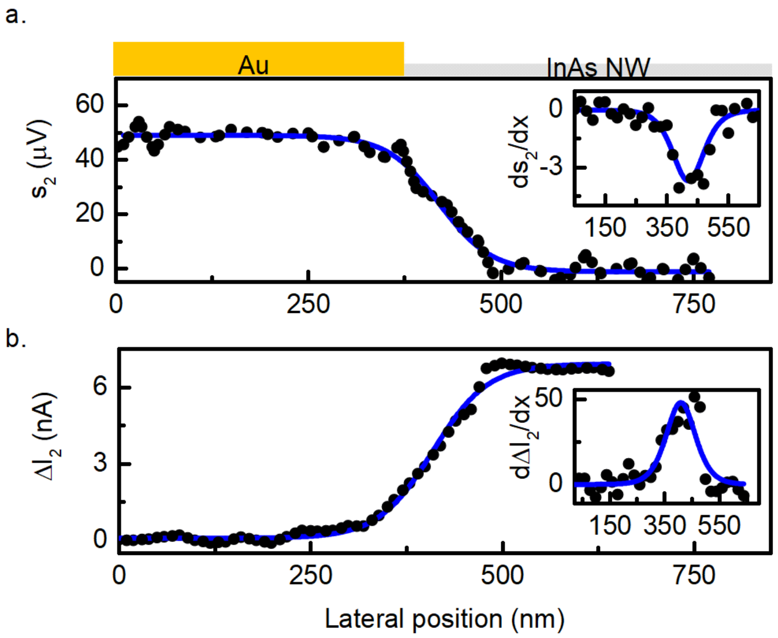

Figure S4: Spatial resolution of s-SNOM measurements. **(a)** THz near-field self-mixing signal at the second harmonics s_2 (black dots) across source-electrode/NW interface together with the best fit using Boltzmann edge function (solid blue line). The first derivative of the signal ds_2/dx (black dots) is reported in the inset together with Lorentzian fit function (blue line) to estimate lateral spatial resolution. **(b)** THz near-field photocurrent ΔI_2 (black dots) across source-electrode/NW interface together with the best fit using Boltzmann edge function (solid blue line); the first-derivative of ΔI_2 is reported in the inset together with the Lorentzian fit function (blue line) to estimate lateral spatial resolution.

2.2 Gate-dependent photocurrent maps at $V_{DS} = 10$ mV

The photocurrent in the NW1 integrated along the nanowire for high bias voltage V_{DS}= 10 mV is reported in Figure S4 as a function of the gate voltage. The profile shows the bolometric fingerprint above the threshold V_G identified in the condition V_{DS}= 1 mV. This is expected because the bolometric photocurrent scales with the applied bias. The broad minimum in the detected photocurrent at the center of the NW1, is an effect of the screening of the THz field operated by the broad gate electrode (1 μm), located at a relatively close (210 nm) distance from the NW axis.

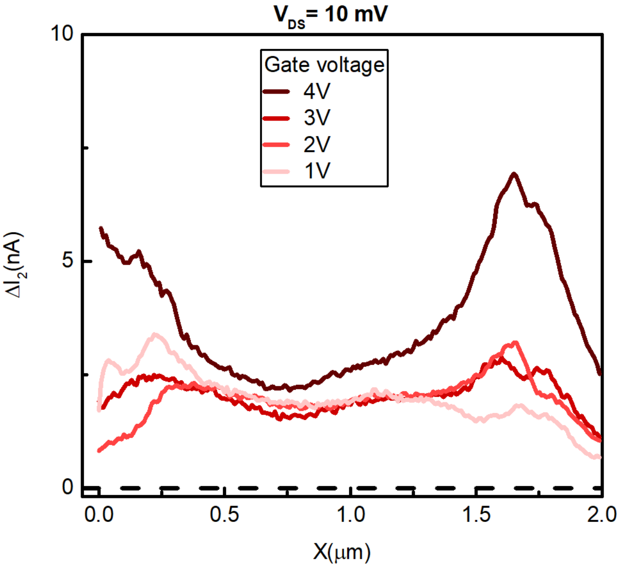

Figure S5 Near-field second order photocurrent ΔI_2 measured along the axis *x* of NW1, at room temperature, while applying a voltage $V_{DS} = 10$ mV and while sweeping the gate voltage V_G in 1-4 V range showing signature of bolometric photocurrent.

2.3 Self-mixing signal as function of the source-drain bias

A negligible variation of second order near-field self-mixing signal s_2 was observed when we change the current flowing in the nanowire by progressively varying V_{DS}, see Fig. S6 for NW1.

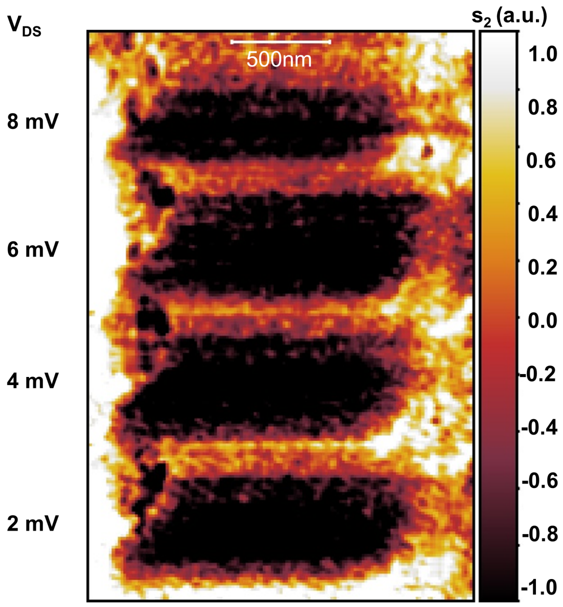

Figure S6 Self-mixing signal s_2 demodulated at the second harmonics of the tapping frequency collected from NW1, for different values of the applied source-drain voltage $V_{DS,}$ and with V_G= 2 V.

Thermal transport in the NW

We use heat transport theory to simulate the lattice T_{ph} and electrons T_e temperature profiles along the NW as a function of the tip position. To this purpose, we first determine the electromagnetic field power density $F(x;\, x_{tip})$ induced by the THz illumination at each tip position. We use tridimensional (3D) FEM simulations with the electromagnetic wave module of COMSOL Multiphysics in the frequency domain. We modeled a cylindrical NW having length of 1.4 μm and a diameter of 80 nm diameter, centered in (x,y,z) = (0,0,0) elongated on the x axis and with the same dielectric properties as NW1 (σ=233 S m^{-1} and ε_r =14.6). The NW is placed in air on the top of a SiO_2 (500 nm)/intrinsic silicon (350 μm) wafer as shown in Fig. S7a. In the 3D model we include the tip as a truncated cone of full height 80 μm and 10° half-angle ended with a semi-spherical apex of 10 nm radius, centered in x_{tip} and with the apex to NW surface distance equals to h. We compute the electric field distribution E and F for two sets of parameters (x_{tip}; h) corresponding to h = 20 nm (simulating the approached tip), and h = 50 nm (simulating the free tip) and x_{tip} ranging from -800 nm to 800 nm in order to include the entire NW and, partially, the electrodes in the scan. The incident *p*-

polarized THz field at 2.7 THz enters from the source contact side with an incidence angle of 30°, as in the experiment.

The coupled differential equations for T_e and T_{ph} describing diffusive heat transport are:

$$\begin{cases} -k_e \frac{\partial^2 T_e}{\partial x^2} + \gamma_{e-ph}(T_e - T_{ph}) = F(x; x_{tip}) \\ -k_{ph} \frac{\partial^2 T_{ph}}{\partial x^2} + \gamma_o(T_{ph} - T_o) = \gamma_{e-ph}(T_e - T_{ph}) \end{cases}$$

where $k_e =$ 0.0017 W K^{-1} $m^{-1}$is the electron thermal conductivity for a NW with σ =233 S m^{-1} according to the Wiedemann-Franz law, $\gamma_{e-ph} = 10^9$ W K^{-1} m^{-3} is the electron-phonon coupling that according to reported similar NW systems[3–5] should be in the range $10^6 \div 10^{13}$ W K^{-1} m^{-3}, $k_{ph} = 1$ W K^{-1} m^{-1} is the lattice thermal conductivity of InAs NWs with 80 nm diameter as experimentally determined[6] and $\gamma_{o/l} = \gamma_o/l$ where $\gamma_o \sim 1$ MW K^{-1} m^{-2} is the out-of-plane thermal conductivity describing the heat transfer from the NW to the substrate, l = 120 nm is the NW effective thickness and F is parametrized in tip position x_{tip}.

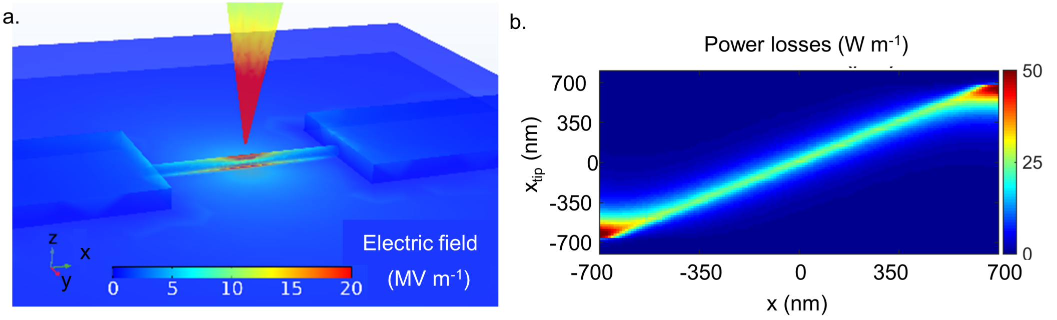

Figure S7 Electric field E distributions computed with Comsol Multiphysics for an incident p-polarized field of 100 μW at a frequency of 2.7 THz. **(a)** zoom of the *E* maps around the NW showing the cylindrical NW, the tip at x_{tip}= 0 nm, the drain and source electrodes and the SiO_2 substrate. **(b)** Power losses linear density as function of the x position along the NW axis and of the tip position x_{tip}, obtained integrating the power losses density F(x,y,z) in the circular cross section of the NW in the zy plane.

To solve the system of differential equations we consider as boundary condition that far from the NW at the electrodes, where F is zero, the temperatures are at equilibrium: T_e (x_{tip}= -800 nm) = T_e (x_{tip}= 800 nm) = T_{ph} (x_{tip}= -800 nm) = T_{ph} (x_{tip}= 800 nm) = T_o.

Near-field photocurrent at higher demodulation order

The s-SNOM signal at demodulation orders higher than n= 2 shows similar spatial distribution both for the photocurrent and for the self-mixing experiment. In Fig. S8, we report the photocurrent maps of NW2 in the PTE (V_G= 6 V) and bolometric (V_G= 0 V) regimes acquired with incident power P= 110 μW at the demodulation orders n= 2-5, while in Fig. S9, we report the corresponding line profiles taken along the dashed lines of Fig. S8 and averaged for 10 pixels (50 nm), around the NW center.

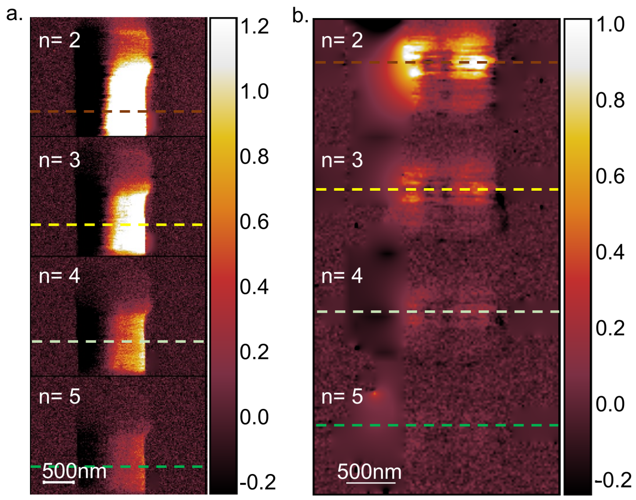

Figure S8. Near-field photocurrent ΔI_n maps in PTE regime **(a)** achieved while applying a gate voltage V_G = 6 V and in the bolometric regime (**b**) with V_G = 0 V as a function of the demodulation order n = 2-5 for NW2 illuminated with incident power P =110 μW.

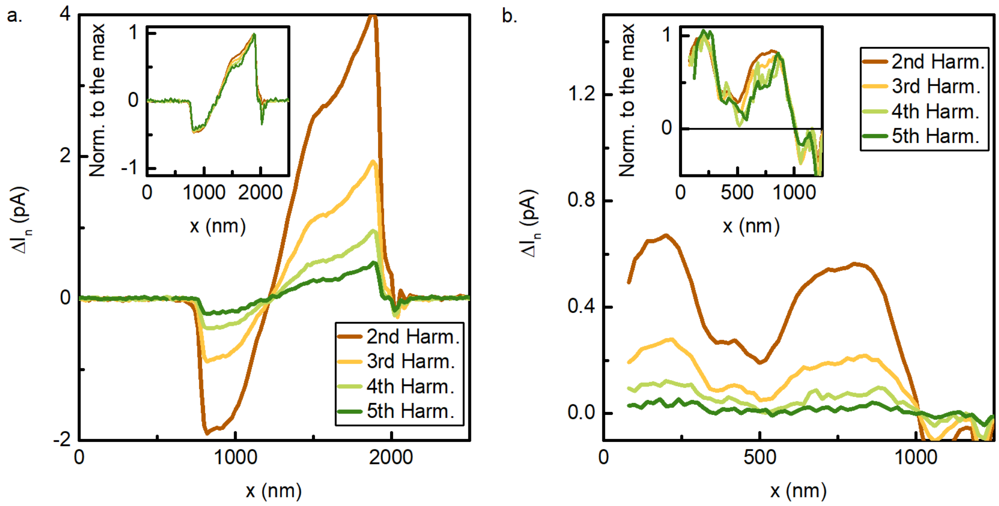

Figure S9. Near-field photocurrent ΔI_n profiles **(a)** in the PTE regime at V_G = 6 V and **(b)** in the bolometric regime at V_G = 0 V, extracted from Fig. S8 along the dashed lines, corresponding to different demodulation harmonics. The curves normalized to the maximum are reported in the related panels inset.

While the intensity of the near-field photocurrent ΔI_n decreases with the demodulation order, as expected[2] (see also Fig. S3), all the harmonics exhibit similar normalized profiles (see the insets).

References

1. Mitrofanov, O. *et al.* Near-field terahertz probes with room-temperature nanodetectors for subwavelength resolution imaging. *Sci. Rep.* 7, 44240 (2017).
2. Knoll, B. & Keilmann, F. Enhanced dielectric contrast in scattering-type scanning near-field optical microscopy. *Opt. Commun.* 182, 321–328 (2000).
3. Sugaya, T. *et al.* Experimental studies of the electron–phonon interaction in InGaAs quantum wires. *Appl. Phys. Lett.* 81, 727–729 (2002).
4. Prasad, C., Ferry, D. K. & Wieder, H. H. Energy relaxation studies in $In_{0.52}Al_{0.48}As/In_{0.53}Ga_{0.47}As/In_{0.52}Al_{0.48}As$ two-dimensional electron gases and quantum wires. *Semicond. Sci. Technol.* 19, S60–S63 (2004).
5. Prasad, C., Ferry, D. K. & Wieder, H. H. Power loss measurements in quasi-1D and quasi-2D systems in an $In_{0.52}Al_{0.48}As/In_{0.53}Ga_{0.47}As/In_{0.52}Al_{0.48}$ Asheterostructure. *J. Vac. Sci. Technol. B Microelectron. Nanom. Struct. Process. Meas. Phenom.* 22, 2059–2062 (2004).
6. Swinkels, M. Y. *et al.* Diameter dependence of the thermal conductivity of InAs nanowires. *Nanotechnology* 26, 385401 (2015).
